# The role of sub-retinal fluid in determining treatment outcomes in patients with neovascular age-related macular degeneration - a phase IV randomised clinical trial with ranibizumab: the FLUID study

**DOI:** 10.1186/s12886-016-0207-3

**Published:** 2016-03-24

**Authors:** Jennifer J. Arnold, Caroline M. Markey, Nicol P. Kurstjens, Robyn H. Guymer

**Affiliations:** Marsden Eye Specialists, 152 Marsden St, Parramatta, NSW 2150 Australia; Markey Medical Consulting Pty Ltd, PO Box 136, Frenchs Forest, NSW 1640 Australia; Novartis Pharmaceuticals Australia, Pty Ltd, 54 Waterloo Rd, Macquarie Park, NSW 2113 Australia; Centre for Eye Research Australia, Royal Victorian Eye and Ear Hospital, University of Melbourne, 32 Gisborne St, East Melbourne, VIC 3002 Australia

**Keywords:** Neovascular AMD, Ranibizumab, Treat and extend regimen, Intra-retinal fluid, Sub-retinal fluid

## Abstract

**Background:**

With increasing experience using anti-VEGF therapy for the treatment of neovascular age-related macular degeneration (nAMD), ophthalmologists have shifted away from a “one size fits all” to an “individualised” approach based on disease activity with the aim of achieving a fluid-free retina. The FLUID study investigates the non-inferiority of a Treat and Extend (T&E) protocol of 0.5 mg ranibizumab, which allows treatment extension in the presence of incomplete resolution of sub-retinal fluid (SRF) ≤200 μm at the foveal centre relative to a T&E protocol that requires complete resolution of all retinal fluid (i.e., both SRF and intra-retinal fluid [IRF]) in patients with nAMD.

**Methods/Design:**

This 24 month, randomised, phase IV trial has completed recruitment of treatment-naïve patients randomised 1:1 to ranibizumab “intensive” treatment (complete resolution of IRF and SRF) or ranibizumab “relaxed” treatment (resolution of IRF or >200 μm SRF only at foveal centre). Patients in both arms follow a T&E regimen where extension decisions are based upon assessment of lesion activity: loss of ≥5 letters of visual acuity, new haemorrhage, presence of IRF and SRF on an optical coherence tomography (OCT) scan. The determination of SRF is conducted at a reading centre while the assessment of IRF is physician-determined. The primary endpoint is the mean change in best-corrected visual acuity (BCVA) from baseline to 24 months. Secondary endpoints include the mean change in central retinal thickness (CRT) from baseline to 12 and 24 months, the number of ranibizumab injections administered at 12 and 24 months, and the pharmacogenomic assessment of AMD Gene Consortium-identified single-nucleotide polymorphisms (SNPs) and their association with treatment response. Three hundred and forty seven (347) patients have been recruited by 16 Australian sites within approximately 16 months. A protocol to adjudicate on SRF has been established by the central reading centre and is demonstrating good concordance with investigator assessment.

**Discussion:**

This study will provide important insights into retreatment criteria for managing nAMD using a T&E regimen. The current paper describes the clinical rationale for using a less intensive treatment approach using ranibizumab and details of the treatment protocol.

**Trial registration:**

Trial registration number: NCT01972789. Date of registration: 24th October 2013.

## Background

The advent of anti-vascular endothelial growth factor (VEGF) designed for intraocular injection has revolutionised the treatment of neovascular age-related macular degeneration (nAMD), the leading cause of severe vision loss in the elderly population. Coupled with diagnostic advances and improvements in monitoring, anti-VEGF therapy has contributed significantly to a decrease in cases of blindness, improvement in patient quality of life, and overall public health expenditure on the consequences of vision loss [1].

The pivotal trials of ranibizumab (Lucentis), MARINA [2] and ANCHOR [3], investigated the efficacy and safety of monthly injections on visual acuity, thus this treatment regimen subsequently formed the basis for its initial registration indication [4]. With the evaluation of different injection regimens through subsequent prospective clinical trials (PIER, EXCITE, SUSTAIN) and increasing clinical experience by ophthalmologists, it became clear that less frequent ranibizumab injections could yield visual benefits in certain subgroups of patients comparable to those of monthly injections [4–10]. In the randomised, controlled trial CATT, which evaluated monthly monitoring and a *pro re nata* (PRN) or ‘as needed’ treatment approach with anti-VEGF therapy, PRN ranibizumab treatment was observed to be non-inferior to monthly ranibizumab in terms of visual outcome at one year, but reduced the number of injections by almost one-half, although the second year results demonstrated a greater gain in visual acuity with monthly injections relative to PRN for both ranibizumab and bevacizumab treatments (*p* = 0.046 for regimen) [11, 12]. A similar outcome to that of the CATT one year results was observed in the HARBOR study, although the pre-specified non-inferiority comparison was not met for the 0.5 mg ranibizumab PRN dose [13]. Individualised approaches, requiring the determination of disease activity, is now widely used in protocols using all anti-VEGF agents.

A modified PRN approach, which is more proactive, is termed Treat and Extend (T&E); with this, patients receive a mandatory injection at each visit but the interval for the subsequent visit is determined by the clinician’s assessment of the disease activity. Generally, the extension interval for such a protocol is 2 weeks and the retreatment criteria is based on, among other morphological and visual indices, resolution of all SRF and IRF. A number of retrospective/observational and prospective studies in which a T&E protocol has been implemented have reported visual gains of, on average, 9.6 letters (range: 7–11 letters across cited studies) in the first year of treatment and 8.7 letters (range: 8–9.2 letters across cited studies) in the second year of treatment [14–18]. These outcomes have been achieved with an average of 8.1 injections in the first year of treatment and 6.3 injections in the second year of treatment. In all studies cited above, patients were treated with ranibizumab 0.5 mg (with the exception of one study in which 27 % patients were treated with bevacizumab) and the physicians all treated with the goal of achieving a dry retina (retinal fluid was a key retreatment criterion). These outcomes are comparable to those described in large, real-life, observational studies of anti-VEGF therapy in nAMD patients [19, 20].

The definition of disease activity has historically been based upon three parameters: a loss of ≥5 letters in visual acuity (VA), evidence of new haemorrhage and the presence of intra-retinal fluid (IRF) and sub-retinal fluid (SRF) as determined on an optical coherence tomography (OCT) scan [21]. Parameters of choroidal neovascularisation (CNV) activity on OCT scanning have developed and refined with improvements in OCT technology and clinical experience. Most individualised or PRN treatment regimens, including those used to guide treatment in the CATT [11, 12] and HARBOR [13] clinical trials plus the T&E studies described above [14–18], aim to completely resolve all IRF and SRF to achieve a “dry” retina as seen on OCT scanning. However, it is not known if all “fluid”, as determined by dark empty spaces on the OCT, truly indicate ongoing disease activity and, as such, whether their presence should always mandate an “active disease” response leading to a change in interval duration.

To date, there is poor correlation between VA outcomes and the fluid status of the retina. The CATT 2 year results revealed that a doubling of the proportion of patients who achieved a dry retina (ranibizumab PRN arm: 22.3 % patients vs. ranibizumab monthly arm: 45.5 % patients) did not equate to any change in the proportion of patients who gained 15 letters or more (ranibizumab PRN arm: 30.7 % patients vs. ranibizumab monthly arm: 32.8 % patients) [12]. Similarly, the 96 week VIEW [22] study results revealed little difference in the proportion of patients who gained 15 letters or more (ranibizumab monthly arm: 34.9 % patients vs. aflibercept monthly arm: 29.4 % patients) or the number of letters gained (ranibizumab monthly arm: 9.4 letters vs. aflibercept monthly arm: 7.6 letters) in spite of a notable difference in the proportion of patients achieving a dry retina (ranibizumab monthly arm: 60.4 % patients vs. aflibercept monthly arm: 80.3 % patients) [22].

Currently, it is not known whether it is imperative to resolve all of both IRF and SRF to achieve best results. It may be possible to allow some SRF to remain and to continue extending the treatment interval without any detriment to the long term visual outcome. It is possible that, in some cases, the SRF simply reflects the topography of the retina and underlying structures where there is a “gap” between peaks of drusen and retinal pigment epithelium (RPE) and the overlying retina; it may not be an indication of ongoing neovascular disease activity. Indeed, a study of 214 eyes that received anti-VEGF therapy for the treatment of nAMD demonstrated that the combined presence of IRF and SRF at baseline lead to worse presenting visual acuity (20/120) compared to the presence of SRF only (20/72). At the end of three consecutive monthly loading doses of anti-VEGF therapy, eyes with residual IRF (in combination with SRF) averaged 20/180 while eyes with no fluid (either SRF or IRF) averaged 20/80 (*p* = 0.05) which was comparable to eyes with SRF alone (20/90). While this study strongly suggests that fluid may be a predictor of visual outcomes in patients treated with anti-VEGF therapy, this does not discount the potential role of other factors, such as fibrosis, atrophy and subretinal hyper-reflective material (SHRM), in predicting response.

One consequence of insisting upon complete lack of IRF and SRF is a greater injection frequency, with the inherent risks associated with injections of drug into the vitreous cavity, both ocular (such as endophthalmitis, lens trauma and retinal detachment) and potentially any systemic events. An additional consequence of greater injection frequency is the potential for earlier progression of macular atrophy (also termed geographic atrophy in the literature), which has been hypothesised as being exacerbated over time with intensive, long-term anti-VEGF therapy. The CATT study [12] 2 year results revealed a 59 % increase in the risk of geographic atrophy with 2 years of monthly injections (average 22.5 injections) regardless of anti-VEGF used in comparison with PRN treatment (average 13.1 injection; *p* = 0.003) [12]. Further, Grunwald et al. [23] demonstrated that eyes exhibiting SRF in the foveal centre were at lesser risk of developing geographic atrophy than those without SRF by 2 years (when subretinal fluid thickness was >25 μm, the aHR was 0.52 (95 % CI, 0.35-0.78)). Conversely, eyes with IRF in the foveal centre (aHR, 2.10; 95 % CI, 1.34-3.31) or away from it (aHR, 1.80; 95 % CI, 1.10-2.95) had a higher risk of developing geographic atrophy compared with eyes with no IRF [23].

What has become increasingly apparent is that patients show an individualised response to therapy and opportunities to further optimise treatment need to be explored and understood. The FLUID study (a phase IV, randomised, controlled, single masked study investigating the efficacy and safety of ranibizumab T&E using an intensive retinal fluid retreatment regimen compared to a relaxed retinal fluid retreatment regimen in patients with nAMD) has been implemented to address these questions. This study will explore a T&E regimen by testing the non-inferiority of a relaxed fluid management approach (tolerance of any SRF unless specifically within subfoveal area in which case only ≤ 200 µm SRF will be tolerated) compared to an intensive fluid management approach (no tolerance of SRF) as measured by the mean change in best-corrected visual acuity (BCVA) from baseline to 24 months. A range of pre-defined secondary endpoints will further our understanding of the impact of variable treatment approaches, as well as determining the influence of genotypes on treatment response and outcomes. While current research is inconclusive as to the association between genotype and treatment response, some studies have demonstrated poorer visual outcome following ranibizumab or bevacizumab therapy in patients expressing the homozygous CC risk genotype at amino acid 402 of the *CFH* gene and HTRA1 promoter SNP (rs11200638) and A69S at LOC387715/ARMS2, as described by Brantley et al. [24] and Abedi et al. [25], respectively.

The study hypothesis is that ranibizumab 0.5 mg when administered to resolve IRF and/or SRF > 200 μm only at the foveal centre (relaxed retinal fluid management) results in visual acuity benefit that is not clinically worse than when administered to completely resolve both IRF and SRF (intensive retinal fluid management) in patients with nAMD.

This paper describes the methodology behind the FLUID study and specific details of the T&E methodology.

## Methods/Design

This is a multi-centre, randomised, two arm study being conducted in 16 sites across Australia (http://clinicaltrials.gov/show/NCT01972789) [26]. A total of 347 nAMD patients have been recruited over an approximate 16 month period (30th October 2013 – 3rd March 2015).

This clinical study was designed, implemented and will be reported in accordance with the International Conference on Harmonization (ICH) Harmonized Tripartite Guidelines for Good Clinical Practice, with applicable local regulations (including European Directive 2001/20/EC, US Code of Federal Regulations Title 21, and Japanese Ministry of Health, Labor, and Welfare) and with the ethical principles laid down in the Declaration of Helsinki. Ethics Approval has been obtained for all sites within this study (Bellberry Limited Human Research Ethics Committee for 13 sites (in New South Wales, Victoria, South Australia, Western Australia, Tasmania), Macquarie University Human Research Ethics Committee for one site (in New South Wales), The Royal Victorian Eye and Ear Hospital Human Research Ethics Committee for one site (in Victoria) and The Alfred Ethics Committee for one site (in Victoria). Patients are enrolled once written informed consent has been obtained from patients by the Principal Investigator or Sub-Investigator following full disclosure of the study and prior to any study related assessment or investigation is initiated.

A protocol amendment was made on 13th September 2013 to implement masking of the central reading centre (being used to adjudicate on fluid status in patients), the visual acuity assessor at study sites, and the patients. A second protocol amendment was made on 11th February 2014 to address administrative anomalies, provide clarification on some aspects of the methodology, implement changes in the timing schedule for which certain study procedures are to be performed, and modify exclusion criteria to allow patients presenting with pseudoexfoliation and add prohibitive treatments to the study eye. A third protocol amendment was made on 10th December 2014 to remove the emergence of the following adverse events – full thickness macular hole, stroke, myocardial infarction, transient ischemic attack and rhegmatogenous retinal detachment – from the list requiring a patient to be discontinued to the list for which ranibizumab treatment be delayed until the condition is successfully treated per investigator discretion. This was implemented to bring the management of patients with these conditions into alignment with standard clinical practice. In addition, the amendment implemented alteration of the procedure relating to the adjudication of retinal fluid so that the sites are able to adjudicate on IRF while the central reading centre continues to adjudicate on SRF (outlined in detail below under the subtitle “Masking”). A fourth protocol amendment was made on 12 October 2015 to clarify that IRF and intraretinal cysts that, in the Investigator’s opinion, are likely to be recalcitrant to anti-VEGF treatment are not considered in the treatment protocol when making treatment extension decisions to align with standard clinical practice. Further, this amendment outlined the availability of ranibizumab in the form of a pre-filled syringe.

The ranibizumab (Lucentis) administered in this study is prescribed and dispensed via the Australian Pharmaceutical Benefits Scheme (PBS) and in compliance with all regulatory and reimbursement requirements.

### Inclusion and exclusion criteria

As outlined in Table 1, patients at least 50 years old with a diagnosis of active subfoveal CNV secondary to nAMD confirmed on fluorescein angiography (FA) in accordance with PBS eligibility criteria and with evidence of fluid on OCT with at least one treatment-naïve eye meeting study criteria who are able to comply with study requirements are being enrolled. Active CNV affecting the fovea was defined on multi-modal imaging as subfoveal CNV (determined on FA) and, in addition, juxtafoveal or extrafoveal CNV where there was a component of the CNV (ie fluid, SHRM) involving the fovea on OCT. The presence of fluid (SRF and/or IRF) in the study eye at baseline is not mandatory for inclusion of patients into the trial.

**Table 1:** Key Inclusion Criteria

1. At least 50 years old.
2. Diagnosis of active subfoveal CNV secondary to nAMD confirmed on FA in accordance with PBS eligibility criteria and with evidence of fluid on OCT. Active CNV affecting the fovea was defined on multi-modal imaging as subfoveal CNV (determined on FA) and, in addition, juxtafoveal or extrafoveal CNV where there was a component of the CNV (ie fluid, SHRM) involving the fovea on OCT.
3. At least one treatment-naïve eye.
4. BCVA score of at least 23 letters or more (Snellen visual acuity or equivalent of 20/320).

While the study eye must be treatment-naïve, the fellow eye may have been, or can be, treated with any anti-angiogenic drug (including any anti-VEGF agents) prior to baseline.

Patients must have a BCVA score of at least 23 letters or more as measured by the 3 m (9.84 ft) Early Treatment Diabetic Retinopathy Study (ETDRS) logarithm of the minimum angle of resolution (logMAR) charts (a Snellen visual acuity or equivalent of 20/320 can be alternatives). There is no upper limit to BCVA eligibility.

The ocular exclusion criteria for the study eye include visually significant cataract, aphakia, severe vitreous haemorrhage, rhegmatogenous retinal detachment, proliferative diabetic retinopathy or CNV of any other cause than nAMD identified at the time of screening or baseline. In addition, any structural damage within 0.5 disc diameter of the centre of the macula (e.g. vitreomacular traction, epiretinal membrane, scar, laser burn, foveal atrophy) at the time of screening that in the Investigator’s opinion could preclude visual function improvement with treatment is an exclusion criterion. Further, patients treated with any anti-angiogenic drugs (including any anti-VEGF agents) prior to baseline are excluded as are those having any intraocular procedure (including Yttrium-Aluminium-Garnet capsulotomy) within 2 months prior to baseline or anticipated within the next 6 months following baseline. Exclusion criteria applicable to both eyes include any active periocular or ocular inflammation/infection, uncontrolled glaucoma on medication or neovascularisation of the iris or neovascular glaucoma.

Patients with cardiovascular disease, including a history of stroke or myocardial infarction less than 3 months prior to screening, or an uncontrolled blood pressure defined as systolic value of >160 mm Hg or diastolic value of >100 mm Hg at screening or baseline cannot be enrolled.

Prohibited treatments include use of intra- or periocular corticosteroids, or intraocular corticosteroid implants, as are use of other investigational drugs and use of systemic medications known to be toxic to the lens, retina or optic nerve as outlined in the Lucentis Australian Prescribing Information [4] at any time during the study.

### Endpoints

All efficacy assessments are performed on the study eye (and, at certain time-points, on the fellow eye) and include both functional and anatomical evaluations. The primary endpoint for the study is the mean change in BCVA from baseline to month 24. The key secondary endpoints are the mean change in BCVA from baseline to month 12, the mean change in central retinal thickness (CRT) from baseline to month 12 and 24, and the mean number of injections from baseline to month 12 and 24. Additional secondary objectives include the proportion of patients showing newly developed macular atrophy at months 12 and 24, mean change in area of new and existing macular atrophy from baseline to month 12 and 24, proportion of patients showing no IRF and SRF at months 2, 12 and 24, proportion of patients showing ≥15 ETDRS letter gain from baseline to month 12 and 24, proportion of patients showing < 15 ETDRS letter loss from baseline to month 12 and 24, number of rebounds back to monthly injections, determination of genotypes associated with AMD or response to treatment (and correlation of these genotypes with VA outcome and ability to dry the retina), proportion of patients with both SRF and IRF who despite monthly treatment do not resolve their SRF as well as ocular and systemic adverse events.

For the assessment of the presence of macular atrophy, a multimodal imaging approach will be used. Image modalities will include fundus autofluorescence (AF) imaging, infrared imaging, OCT and colour fundus (CF) photographs. Atrophy will be diagnosed if FA and one other modality confirm the presence of macular atrophy.

For the determination of genotypes associated with the response to treatment, this will be made not only in terms of VA achieved and gain or loss of ETDRS letters at month 12 and 24, but also in terms of the ability to dry the retina of either (or both) IRF and SRF after the first three monthly treatments of ranibizumab or after the forth treatment of ranibizumab (the first possible time for extension).

### Treatment assignment and assessment

**Fig. 1:**
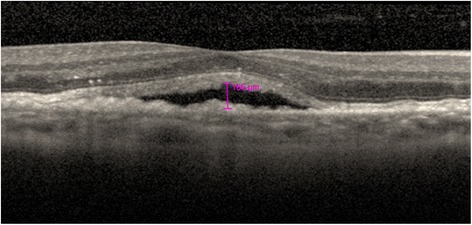
OCT image showing presence of SRF (<200 μm height) within the subfoveal region of the macula that constitutes disease activity in the Intensive retinal fluid arm only

At baseline, patients were randomised 1:1 via an Interactive Web-based Response System (IWRS) to either an:

- Intensive retinal fluid treatment regimen (following the initial 3 monthly injections of ranibizumab 0. 5 mg, treatment interval is determined by disease activity, aiming for complete resolution of all retinal fluid), or
- Relaxed retinal fluid treatment regimen (following the initial 3 monthly injections of ranibizumab 0. 5 mg, treatment interval is determined by disease activity, aiming for resolution of only IRF, and/or ≤200 µm of SRF; Fig. 1).

The IWRS (Balance) is integrated with the electronic data capture (EDC) system (Medidata) and therefore site personnel (Principal Investigator, Sub-Investigator or Study Coordinator) access the IWRS through the EDC system. Patient randomisation number and assignment to treatment arm are provided by this IWRS/EDC system.

For both treatment groups, all patients receive three monthly injections of ranibizumab (0.5 mg in 0.05 mL) at baseline, week 4 and week 8. Following the third injection, the patient is assessed for disease activity in the study eye as set out in Table 2 and determination of treatment interval is made.

**Table 2:** Criteria for Disease Activity

1. A loss of VA ≥5 letters than the best VA recorded since baseline.
2. New retinal haemorrhage.
3. The presence of IRF or SRF on OCT.
For patients in the Intensive retinal fluid arm, this criterion is “the presence of any IRF or SRF”.
For patients in the Relaxed retinal fluid arm, this criterion is “the presence of any IRF or SRF >200 μm in height at subfoveal centre” (as measured by the caliper function on the OCT). Sub-RPE fluid does not constitute part of the decision-making criteria for either arm.

### Treatment extension

In both arms, if any of these signs of activity are present in the study eye, either singly or in combination, the subsequent injection visit interval is kept at 4 weeks (i.e. 28 day interval; no increase in the treatment interval). If none of these signs are present, the subsequent injection interval is extended by 2 weeks, i.e. out to 6 weeks. This process continues with 2 week extensions, as long as there is no return of disease activity, until the maximum retreatment interval of 12 weeks is reached. Once treatment interval extension has occurred, the presence of disease activity in the study eye requires a reduction in the treatment interval as follows:

- If one sign of disease activity, as defined above, is seen and the patient is currently on a 4 weekly interval then the interval remains at 4 weeks.
- If one sign of disease activity, as defined above, is seen and the patient is currently on a >4 weekly interval then the interval is reduced by 2 weeks.
- If two (2), or more, signs of disease activity, as defined above, are seen then the interval of injections reverts back to 4 weekly injections.

The interval is further decreased by 2 weeks (if the interval is not currently at 4 weeks) at the subsequent visit(s) if signs of activity continue to be detected using this approach. Extension will again occur at a subsequent visit if there are no signs of disease activity. However, after the second attempt at extension, any further attempts will have the maximum interval of 2 weeks less than the interval at which activity previously re-occurred (referred to as the break point). If the patient was returned to 4 weekly intervals on the first extension and again on the second extension, then no more attempts at extension will occur during the study.

To align with real-world scenarios typical in standard clinical practice, there are no specific withdrawal criteria that relate to vitrectomy or other surgical intraocular procedures (other than use of anti-VEGF, corticosteroid or investigational drugs/interventions) once ranibizumab treatment has commenced. Full thickness macular hole, stroke, myocardial infarction, transient ischemic attack and rhegmatogenous retinal detachment require a delay in ranibizumab treatment until the condition is successfully treated per investigator discretion.

### Protocol visits

Patients will undergo assessment of BCVA and OCT at all visits in the study eye. BCVA with refraction and both CF and FA will be performed only at screening/baseline, month 12 and month 24 visits in the study eye (in addition, BCVA with refraction will be performed at the month 2 visit). These same assessments will occur in the fellow eye at screening/baseline, month 12 and month 24 only. The FA images are evaluated by the central reading centre to determine the presence and type of AMD and area of leakage of the CNV.

VA is performed using a logMAR chart at a distance of 3 m at all visits (this distance was specifically chosen to accommodate the average room size for many sites). If it is not possible to perform a subjective refraction or VA testing due to any reason, the refraction/visual acuity testing is attempted at a distance of 1 m. OCT images are taken at all visits after the BCVA assessment and before any injection procedure using High Definition (HD)/Spectral Domain (SD) OCT equipment. AF is optional at sites based on availability of equipment at each site and is performed to measure the presence of existing macular atrophy in the study at screening/baseline and week 8, and the expansion of existing macular atrophy and newly developed macular atrophy at month 12 and 24. For all procedures, the relevant investigational site personnel are certified by the accredited vendors (Klinitrial for VA and the Bern Photographic Reading Center for OCT, FA, CF, AF) prior to commencement of assessments.

The maximum number of patient visits is 26 (assuming monthly visits throughout and separate screening and baseline visits) while the minimum number of patient visits is 13 (assuming extension to 12 weeks with no breaks and separate screening and baseline visits). After completion of the study, patients will return to the standard care of each clinician.

### Masking

To ensure that the primary endpoint measurement of BCVA score is not influenced by bias, both the BCVA assessor and the patient are masked to the treatment assignment. Further, to avoid the introduction of Investigator bias in making retreatment decisions (since the Investigator is both the assessor and injector at the majority of sites), a central reading centre (Bern Photographic Reading Center, Bern, Switzerland) is employed to mask the study and facilitate compliance of Investigator sites to the protocol. The central reading centre is masked to the randomisation and treatment of the patient. All images collected during the study (OCT, FA, CF, AF) are sent to the central reading centre for analysis. Since the primary aim of the study is to investigate whether retreatment should be based on SRF, masked reading centre assessment of the presence of SRF overrides investigator assessment in determining retreatment interval. While the reading centre will read the IRF component as well, the investigator is allowed discretion if they disagree with the reading centre's interpretation of the IRF component. The adjudication of IRF by the investigator site was implemented by a protocol amendment (3) on 10th December 2014 to ensure greater alignment with standard clinical practice since decisions on IRF often cannot be made in isolation (as is the case with a central reading centre assessment) i.e. requiring more than OCT plus an assessment of the patient’s previous response to anti-VEGF therapy to determine if spaces on the OCT are indicative of active disease or, alternatively, if they represent chronic atrophic changes. This change does not impact the key study question, which is whether SRF needs to be completely resolved in making treatment extension decisions since the adjudication of SRF remains with the central reading centre. The original protocol (and protocol amendments 1 and 2) required that IRF adjudication be performed only by the masked reading centre.

### Statistical analyses

The sample size for non-inferiority is based on a one-sided *t*-test with α = 0.025 and 80 % power. For the non-inferiority estimate, the ranibizumab T&E relaxed fluid management arm will be assessed against the ranibizumab T&E intensive fluid management arm and considered non-inferior if the lower limit of the 95 % two sided confidence interval for the difference in changes from baseline BCVA is greater than the negative margin; i.e. the lower limit of the 95 % confidence interval for the difference in change in BCVA score is greater than - 5. This non-inferiority margin of 5 BCVA letters is consistent with that used previously in the landmark CATT study which investigated the effects of anti-VEGF therapies on nAMD [11] and represents a clinically meaningful change in vision. Applying a standard deviation of 15 letters and accounting for a 15 % drop out rate during the 24 month study duration (both used in the CATT study [11]), the minimum number of patients required per arm for mean change in BCVA score is 165 patients. Unless otherwise specified in the Statistical Analysis Plan, for which greater details will be provided in subsequent publication of the FLUID results, the intent-to-treat principle will be used for the primary, secondary and pre-specified exploratory efficacy analyses.

**Fig. 2:**
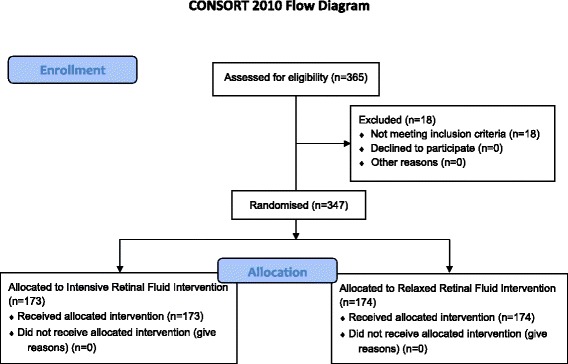
Flow chart outlining patient enrollment and allocation for study

The FLUID study is fully recruited with a total of 347 patients having been recruited in 16 Australian sites located throughout New South Wales, Victoria, Tasmania, South Australia and Western Australia. One hundred and seventy three (173) patients were randomised to the Intensive retinal fluid arm and 174 patients were randomised to the Relaxed retinal fluid arm. Enrolment commenced on 30th October 2013 and was completed on 3rd March 2015 (~16 month period) (Fig. 2). Full data analysis will be reported by the end of 2017. Baseline demographic outcomes and primary and secondary outcomes will be presented in future publications.

## Discussion

Globally, over 90 % of retinal specialists use an individualised approach (either PRN or T&E) in treating their nAMD patients with anti-VEGF therapy [19]. As such, each physician is required to make a judgment on the activity of the CNV lesion. The current best clinical practice states that fluid on an OCT will be considered as an indication of active disease. However, we know that physicians add their own interpretation to the appearance of fluid and make a judgment call on its relevance to disease activity. For example, they question whether a cyst is a chronic atrophic cyst, or whether SRF is simply a failure of the interdigitating photoreceptors and RPE to reconnect tightly thus leaving a small “space”. All of these anatomical features would be interpreted by a reading centre as evidence of fluid and therefore disease activity yet, in many instances, clinicians would not act on these findings. Indeed, in the CATT study, there was a discrepancy of fluid interpretation between the reading centre and investigators in 31 % of adjudications in which the reading centre found fluid where the clinician did not (and thus did not treat with anti-VEGF therapy) [12].

Therefore, there appears to be an “art” based on years of experience that informs a clinician as to the relevance of certain appearances of fluid and how it has behaved with previous treatments when making their decision to treat in the case of PRN protocols, or extend an interval in the case of T&E protocols. Our research in the FLUID study aims to address these unscripted decisions in order to help all clinicians who are now attempting to make judgments on disease activity for their patients presenting with nAMD. The key question being addressed in the study is whether or not a small amount of SRF (≤200 μm) still present at the end of the often-implemented three initial monthly injections is a variable that affects final VA or, alternatively, if remaining SRF is not associated with worse visual outcomes. Note that the figure of 200 μm was chosen as the upper limit of tolerance for SRF by the study Steering Committee who made the clinical judgement that an accumulation of SRF less than this was acceptable to investigators to not actively treat. In addition, 200 μm was deemed sufficiently large to be reproducibly measured by both site and the central reading centre. If the study does not demonstrate any difference in visual outcomes between the two arms, that is, if there is no difference in VA at 24 months between the relaxed fluid treatment regimen compared to the intensive fluid treatment regimen, then it would suggest that clinicians may adopt a treatment approach in which less ranibizumab injections are required to still achieve optimal visual outcomes. It will also highlight the notion that a clinician’s experience in interpreting OCT findings is a crucial factor in achieving optimal outcomes for that individual patient. The more we can articulate what this experience tells us by way of protocols, the easier it will be for clinicians with less experience in this disease to achieve optimal visual outcomes while minimising treatment burden.

## Abbreviations

AF: autofluorescence
AMD: age-related macular degeneration
BCVA: best-corrected visual acuity
CATT: comparison of AMD treatments trials
CF: colour fundus
CNV: choroidal neovascularisation
CRT: central retinal thickness
ETDRS: early treatment diabetic retinopathy study
FA: fluorescein angiography
FRB!: Fight Retinal Blindness!
HD: high definition
ICH: International Conference on Harmonization
IRC: intra-retinal cysts
IRF: intra-retinal fluid
logMAR: logarithm of the minimum angle of resolution
nAMD: neovascular age-related macular degeneration
OCT: optical coherence tomography
PBAC: Pharmaceuticals Benefits Advisory Committee
PBS: pharmaceutical benefits scheme
PRN: *pro re nata*
RPE: retinal pigment epithelium
SD: spectral domain
SNPs: single-nucleotide polymorphisms
SRF: sub-retinal fluid
T&E: treat and extend
VA: visual acuity
VEGF: vascular endothelial growth factor
